# An MRI Scans-Based Alzheimer’s Disease Detection via Convolutional Neural Network and Transfer Learning

**DOI:** 10.3390/diagnostics12071531

**Published:** 2022-06-23

**Authors:** Kwok Tai Chui, Brij B. Gupta, Wadee Alhalabi, Fatma Salih Alzahrani

**Affiliations:** 1 Department of Electronic Engineering and Computer Science, School of Science and Technology, Hong Kong Metropolitan University, Hong Kong, China; 2 International Center for AI and Cyber Security Research and Innovations & Department of Computer Science and Information Engineering, Asia University, Taichung 41354, Taiwan; 3 Lebanese American University, Beirut 1102, Lebanon; 4 King Abdulaziz University, Jeddah 22254, Saudi Arabia; 5 Department of Computer Science, King Abdulaziz University, Jeddah 22254, Saudi Arabia; wsalhalabi@kau.edu.sa; 6 Immersive Virtual Reality Research Group, King Abdulaziz University, Jeddah 22254, Saudi Arabia; 7 Pediatric Department, King Abdulaziz University, Jeddah 22254, Saudi Arabia; falzahrani@kau.edu.sa

**Keywords:** Alzheimer’s disease, automatic diagnosis, convolutional neural network, deep learning, dementia, generative adversarial network, imbalanced dataset, MRI scans, transfer learning

## Abstract

Alzheimer’s disease (AD) is the most common type (>60%) of dementia and can wreak havoc on the psychological and physiological development of sufferers and their carers, as well as the economic and social development. Attributed to the shortage of medical staff, automatic diagnosis of AD has become more important to relieve the workload of medical staff and increase the accuracy of medical diagnoses. Using the common MRI scans as inputs, an AD detection model has been designed using convolutional neural network (CNN). To enhance the fine-tuning of hyperparameters and, thus, the detection accuracy, transfer learning (TL) is introduced, which brings the domain knowledge from heterogeneous datasets. Generative adversarial network (GAN) is applied to generate additional training data in the minority classes of the benchmark datasets. Performance evaluation and analysis using three benchmark (OASIS-series) datasets revealed the effectiveness of the proposed method, which increases the accuracy of the detection model by 2.85–3.88%, 2.43–2.66%, and 1.8–40.1% in the ablation study of GAN and TL, as well as the comparison with existing works, respectively.

## 1. Introduction

As a result of the deterioration in cognitive function, such as impaired ability to make decision, calculate, comprehend, think, and remember, dementia causes the progressive damage of patients’ bodies and brains, and leads to death eventually. The World Health Organization (WHO) has published a document named Global Action Plan on the Public Health Response to Dementia 2017–2025 [1]. Yet, more than 55 million patients are living with dementia, where 60–70% of them suffer from Alzheimer’s disease (AD). Seven action areas were defined as global targets in dementia research and innovation; information systems for dementia; support for dementia carers; dementia diagnosis, treatment, care, and support; dementia risk reduction; dementia awareness and friendliness; and dementia as a public health priority. In each year, there are about 9.9 million new cases [2,3]. It was estimated that dementia may lead to an annual loss of 1.1% (USD 230 billion) of world gross domestic product and was projected to reach USD 2 trillion by 2030. COVID-19 has been challenging on the E-health care [4], disease causality knowledge [5], and point-of-care [6] for AD patients.

To meet the requirement of 4.45 medical staff per 1000 population, it is desired that there will be 16–19 million more health workers by 2030 [7]. Nevertheless, the goal seems not to be achievable in this decade based on the historical records that the global number of medical staff remains steady. In this paper, automatic diagnosis of AD via machine learning model is considered. It not only reduces the workload in medical diagnosis, but also increases the detection accuracy (given that sufficient training data are available). To align with the data type for formal medical diagnosis of AD, MRI scans are considered.

In this section, related works are firstly presented to cover the methodology and results of the existing works. This is followed by the summary of the limitations of the existing works, which are served as the rationale of our proposed algorithm. The last part of Section 1 summarizes the research contributions of our work. In Section 2, datasets and methodology will be presented. An ablation study of GAN and TL will be presented in Section 3. The performance analysis of our work and the comparison between our work and existing works will be detailed in Section 4. Finally, a conclusion is drawn in Section 5.

### 1.1. Related Works

Three benchmark datasets were selected for the performance evaluation and analysis of the AD detection model using MRI scans as inputs. They form three series of the Open Access Series of Imaging Studies, namely OASIS-1 [8], OASIS-2 [9], and OASIS-3 [10]. The details of these datasets will be presented in Section 2.1. The discussion of the methodology and results of the existing works is separated based on each dataset. The works are [11,12,13,14] for OASIS-1, [15,16,17,18] for OASIS-2, and [19,20,21,22] for OASIS-3.

#### 1.1.1. AD Detection Models Using OASIS-1

In [11], researchers built a three-class (healthy, very mild dementia, and mild-to-moderate dementia) AD detection model using gradient boosted random forest and ResNet-50. Accuracy rates of 91.3% and 98.99% were obtained, respectively, which suggested ResNet-50 outperformed the gradient boosted random forest algorithm. Another work [12] considered a binary (healthy and unhealthy) AD detection model. BrainNet2D and BrainNet3D were proposed using 2D slice level and 3D subject level, respectively. Their corresponding accuracies were 79% and 80%. A subsect of OASIS-1 was chosen for implementation of a binary AD detection model [13]. Adversarial autoencoder was applied to reconstruct the healthy samples, which resulted in an enhancement on the specificity (78%) of the model while deteriorating the sensitivity (67%). In [14], a subset of OASIS-1 using balanced healthy and AD classes was used. Three algorithms, namely M-Net_acs_32, M-Net_entropy_32, and M-Net-axial_32, were proposed, which yielded accuracy rates of 71%, 72%, and 74.9%, respectively.

#### 1.1.2. AD Detection Models Using OASIS-2

In regard to the existing works that utilized OASIS-2, a binary AD detection model was built using the Boruta algorithm as feature extraction and deep neural network as classification [15]. Results revealed that the model achieved a sensitivity of 88.2% and a specificity of 100%. A subset of OASIS-2 was selected in [16] to build a binary AD detection model using 3D convolutional neural network (CNN). The accuracy of the model was reported as 97%. In [17], a binary AD detection model was constructed using a support vector machine. The feature vector was based on subject ID, clinical dementia ratio, mini-mental state examination, age, magnetic resonance delay, and normalized whole-brain volume. The achieved accuracy was 68.8%. A voxel-sized independent neural network was used to build a binary AD detection model [18]. A subset of OASIS-2 was used for a preliminary study, where an accuracy of 88.2% was concluded.

#### 1.1.3. AD Detection Models Using OASIS-3

The latest series of the dataset, OASIS-3, received much attention due to the availability of more data compared with OASIS-1 and OASIS-2. In [19], a gray-level co-occurrence matrix was applied to extract features of the MRI scans from a subset of OASIS-3 that was fed into a CNN classifier for a binary classification. The model achieved an accuracy of 90.3%. Ensemble learning was applied to merge four common models, namely Inception-v3, DenseNet121, ResNet50, and ResNet18 [20] for four-class AD detection. It achieved accuracies of 91.4% for normal class and 80.7%, 86.0%, and 88.0% for the other three AD classes. In [21], a four-class AD detection model was constructed. Vertex-based graph CNN was proposed for feature extraction, where the outputs were used as inputs for a recurrent neural network predictor. Performance evaluation of the model showed an accuracy of 82.6%. Researchers in [22] formulated the AD detection problem as anomaly detection, as OASIS-3 consists of normal class as the majority class. Deep convolutional generative adversarial network with encoder was proposed for the AD detection. It achieved an accuracy of 74.4%.

### 1.2. Limitations of the Related Works

The key limitations observed in literature are summarized as follows.

- Only a portion of the dataset was considered in the model implementation and performance analysis in works [13,14,16,18,19];
- Some of the existing works did not employ cross-validation [13,15,16,17,18,19,20,21,22] and improperly defined the ratio of cross-validation [11] in the performance evaluation and analysis of the AD detection models. The trained models without cross-validation may not be designed with optimal sets of hyperparameters and may be more prone to model overfitting;
- The classes in the datasets were regrouped and the total number was reduced in works [11,12,13,14,15,16,17,18,19,22];
- Imbalanced classification was reported in some works [13,15,20] and the remaining works reported the overall accuracies;
- Combing the abovementioned limitations, there is room for improvement in the AD detection models for all series of OASIS datasets.

### 1.3. Research Contributions of Our Work

A three-tier algorithm is proposed by utilizing generative adversarial network, convolutional neural network, and transfer learning (GAN-CNN-TL) to resolve the key limitations of the existing works shared in Section 1.2. The research contributions of our work are highlighted as follows.

- The three series of OASIS datasets (OASIS-1, OASIS-2, and OASIS-3) can be considered as heterogeneous datasets that share similar domain knowledge. Therefore, transfer learning (TL) is proposed to borrow the knowledge from two trained models to fine-tune the hyperparameters in the designated model;
- Generative adversarial network (GAN) is used to generate additional training data in the minority class, moderate dementia, which has only two and three samples in OASIS-1 and OASIS-2, respectively. Therefore, it facilitates the formulation of the AD detection problems as usual k-fold cross-validation;
- Compared with existing works, our work enhances the accuracy of the AD detection model by 1.8–40.1%, using three benchmark datasets.

Apart from these, it is worth noting that there are some important considerations to enhance the validity of the performance evaluation and analysis of the AD detection models with the consideration of full datasets, alignment of the original class labels, and fivefold cross-validation.

## 2. Datasets and Methodology

The details of the three benchmark datasets OASIS-1 [8], OASIS-2 [9], and OASIS-3 [10] are firstly summarized. In regard to the methodology of the AD detection model, it is comprised of three algorithms, including GAN, CNN, and TL. 

### 2.1. Benchmark Datasets

The number of participants, class labels, and number of samples in each class are summarized in Table 1 for OASIS-1, OASIS-2, and OASIS-3. The total samples in each group are 434, 373, and 2168 for OASIS-1, OASIS-2, and OASIS-3, respectively. 

It can be seen from Table 1 that the number of samples drops with the increasing severity of the dementia. In addition, there are imbalanced ratios between the normal class and three types of ADs. Table 2 presents the imbalanced ratios (referenced to the majority class, i.e., Class 0) across different classes in each benchmark dataset. The issue of imbalanced datasets is most severe in OASIS-1, followed by OASIS-2 and OASIS-3. In the literature, it was well demonstrated that the machine learning model will tend to bias towards the majority classes (yielding a better performance) in highly imbalanced datasets [23,24,25]. Downsampling of the majority classes is not chosen because it scarifies the availability of raw (ground truth) samples [26,27,28]. Instead, generating additional samples in minority classes is more appropriate and has been employed in our research study. Data were only generated for classes 2 and 3 where they were minority classes in all datasets. Many research studies [29,30,31] have confirmed the effectiveness of GAN in generating additional training data. 

### 2.2. Overview of the Proposed Algorithm

**Figure 1:**
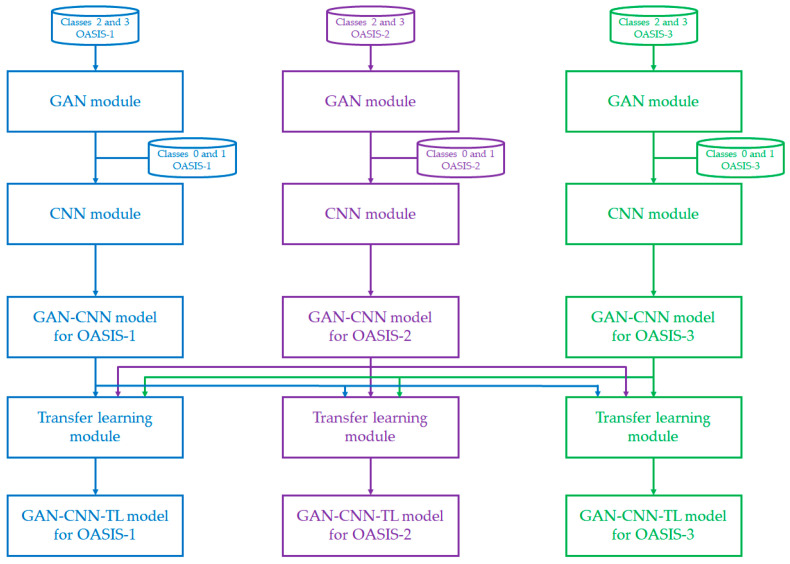
Conceptual diagram of the proposed GAN-CNN-TL algorithm for AD detection.

Figure 1 shows the conceptual diagram of the proposed AD detection algorithm, namely GAN-CNN-TL. The core of the algorithm is based on three modules: GAN module, CNN module, and TL module. As mentioned above, classes 2 and 3 are minority classes in the datasets, so they will pass into a GAN module for additional data generation. The output of this module, along with the remaining two classes (Classes 0 and 1), will serve as the inputs of the CNN module. The CNN module not only extracts features from the MRI scans but also builds an initial AD detection model (GAN-CNN model). A trained (initial) AD detection model of individual datasets will be fine-tuned using transfer learning from two other trained models of other datasets. Here are the illustrations of the three scenarios: (i) a trained GAN-CNN model for OASIS-1 will be fine-tuned using transfer learning from the trained GAN-CNN models for OASIS-2 and OASIS-3; (ii) a trained GAN-CNN model for OASIS-2 will be fine-tuned using transfer learning from the trained GAN-CNN models for OASIS-1 and OASIS-3; and (iii) a trained GAN-CNN model for OASIS-3 will be fine-tuned using transfer learning from the trained GAN-CNN models for OASIS-1 and OASIS-2. As a result, three models are constructed and fine-tuned for each of the OASIS-1, OASIS-2, and OASIS-3. It is noted that the transfer learning is a two-round process, where the hyperparameter tuning will be executed based on one other trained GAN-CNN model at one time. It could reduce the chance of overfitting and computational power.

### 2.3. Additional Data Generation Using GAN

**Figure 2:**
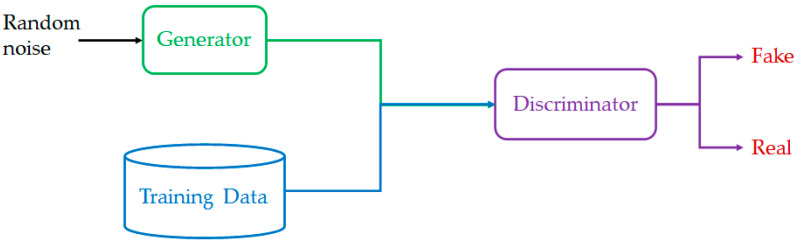
General architecture of GAN.

The general architecture of GAN is shown in Figure 2. Random noise is passed into the generator. The outputs of the generator join the real MRI scans and serve as inputs for the discriminator. The discriminator will determine whether the outputs of the generator can be classified as real (indeed the outputs may fake the discriminator). To begin with, the generator starts generating poor MRI scans, which will be classified as fake data by the discriminator. The experience gained by the generator will enhance the quality of data generation, where the generated data are close to real data. Therefore, the discriminator considers the generated data as real. It is noted that the generated data are not equal to the ground truth data from the training dataset, otherwise, data generation becomes not useful.

The formulations of the GAN are based on two loss functions, namely discriminator loss $L_{D}$ and generator loss $L_{G}$, where D is discriminator, G is generator, $P_{train}\left(x\right)$ is data distribution of training data, $P_{n}\left(n\right)$ is the data distribution of random noise, and $P_{gen}\left(x\right)$ is data distribution of generated data.

The discriminator loss and generator loss of individual samples are given by:

(1) $$L_{D}=\max\left[\log\left(1-D\left(G\left(n\right)\right)\right)+\log\left(D\left(x\right)\right)\right]$$

(2) $$L_{G}=\min\left[\log\left(1-D\left(G\left(n\right)\right)\right)+\log\left(D\left(x\right)\right)\right]$$

Joining these losses, we have the updated loss function as:

(3) $$L_{D,G}=\underset{G}{\min}\underset{D}{\max}\left[\log\left(1-D\left(G\left(n\right)\right)\right)+\log\left(D\left(x\right)\right)\right]$$

To generalize the loss function to the dataset, the GAN algorithm is used to solve the below loss function:

(4) $$\begin{array}{cc} \underset{G}{\min}\underset{D}{\max}F\left(D,G\right) & \\ & =\underset{G}{\min}\underset{D}{\max}(E_{x\sim P_{train\left(x\right)}}\left[\log\left(D\left(x\right)\right)\right]\\ & +E_{n\sim P_{n}\left(n\right)}\left[\log\left(1-D\left(G\left(n\right)\right)\right)\right])\; \end{array}$$

### 2.4. Initial AD Detection Model Using CNN

**Figure 3:**
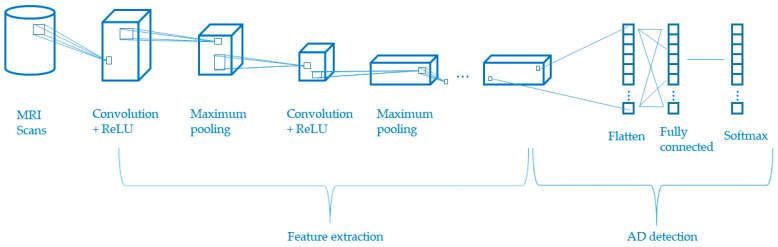
Feature extraction and AD detection using CNN.

The CNN algorithm is adopted to extract feature and build an AD detection model. The raw and generated data from the output of the GAN module are passed into the CNN module. Figure 3 shows the general architecture of the CNN module for feature extraction and AD detection. The major components of CNN include three layers (convolutional layers, maximum pooling layers, and fully connected layers) and three techniques (rectified linear unit (ReLU), image flattening, and softmax activation). Given that the focus of this paper is to enhance the trained GAN-CNN AD model by fine-tuning of the model’s hyperparameters using TL, only the roles of these components are briefly summarized.

- Convolutional layers: The layer comprises filters, feature maps, and zero padding. The filters are considered as the neurons of the layer. The output of a filter employed in the previous layer is called feature map. In some situations, the division between the size of the filter and the size of the previous layer may not be possible and zero padding is needed to ensure the problem is divisible;
- Maximum pooling layers: The layer downsamples the feature map. Mainly, it facilitates the generalization of the feature representation and the reduction of model overfitting;
- Fully connected layers: these are equivalent to the feed-forward neural network layer;
- ReLU: It serves as a piecewise linear function. There are two possible outputs: (i) for negative input, zero will be the output, and (ii) for positive input, the output equals the input;
- Image flattening: the square feature map is flattened and passes into the fully connected layer;
- Softmax activation: facilitates the output of the probability of the class label.

### 2.5. Fine-Tuning the Hyperparameters of the AD Detection Model Using Transfer Learning

**Figure 4:**
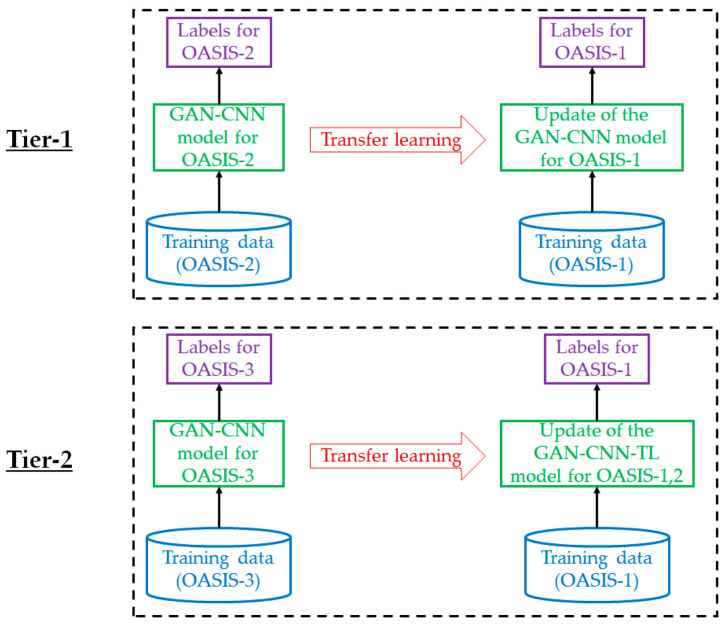
Two-tier transfer learning process with domain knowledge transfer to model for OASIS-1 by OASIS-2 and OASIS-3.

For each of the OASIS datasets, domain knowledge will be transferred from two other OASIS datasets. Therefore, the fine-tuning of the hyperparameters of a trained AD detection model is a two-tier process (Figure 4). For instance, the trained GAN-CNN model for OASIS-1 is fine-tuned using transfer learning from another trained GAN-CNN model for OASIS-2. Afterwards, the target model for OASIS-1 is further fine-tuned using the trained GAN-CNN model for OASIS-3. The idea can be applied to construct the other two models—GAN-CNN-TL model for OASIS-2 and GAN-CNN-TL model for OASIS-3.

The workflows of the transfer learning process are summarized as follows.

- Step 1: begin with a trained GAN-CNN model in dataset 1;
- Step 2: fix the hyperparameters in the lower convolutional layers of the model;
- Step 3: introduce a customized classifier with some layers of tunable parameters to the model;
- Step 4: train the layers with training data;
- Step 5: fine-tuning of the hyperparameters and relaxing some layers if required (for better performance);
- Step 6: repeat steps 1–5 using another dataset.

## 3. Ablation Study

### 3.1. Ablation Study of GAN

To facilitate the optimization of the hyperparameters and examine model overfitting, k-fold cross-validation with k = 5 is adopted [32,33,34].

**Figure 5:**
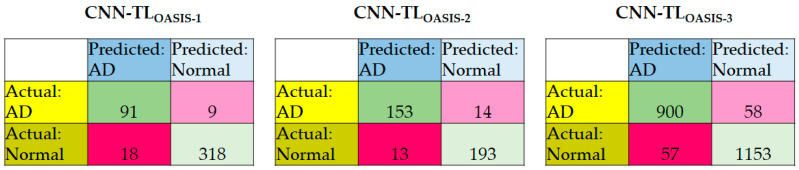
Confusion matrices of the CNN-TL_OASIS-1_, CNN-TL_OASIS-2_, and CNN-TL_OASIS-3_.

Comparison is made between the GAN-CNN-TL and CNN-TL algorithms. It is worth noting that the number of samples in Class 3 in OASIS-1 and OASIS-2 are 2 and 3, respectively. Therefore, the k-fold cross-validation is adjusted to 2-fold cross-validation and 3-fold cross-validation for these datasets, respectively. Table 3 summarizes the performance evaluation of CNN-TL for OASIS-1, OASIS-2, and OASIS-3. Figure 5 summarizes the confusion matrices of the models using three datasets.

The sensitivity and specificity of the model are given by [35]:

(5) $$Sensitivity=\frac{TP}{FN+TP}$$

(6) $$Specificity=\frac{TN}{FP+TN}$$

where *TP* is the true positive, *FN* is the false negative, *TN* is the true negative, and *FP* is the false positive.

- In all models, the specificity is higher than the sensitivity due to the main reason of majority class (Class 0). The accuracy is in between the sensitivity and specificity as the weighted accuracy;
- The ranking in descending order of the sensitivity, specificity, and accuracy of the three models is CNN-TL_OASIS-3_, CNN-TL_OASIS-1_, CNN-TL_OASIS-2_;
- There are deviations between the accuracies of a single class because of the number of available ground truth data in each class and the characteristics (and thus the difficulty in AD detection) of the class. The average deviations are 32.3%, 15.8%, and 1.60% for CNN-TL_OASIS-1_, CNN-TL_OASIS-2_, and CNN-TL_OASIS-3_, respectively. This reflects the fact that GAN is important to generate additional training data in minority classes (Class 2 and Class 3) in order to reduce the impact of biased detection towards the majority class (Class 0);
- The deviations between the sensitivity and specificity are 3.96%, 2.29%, and 1.28% for CNN-TL_OASIS-1_, CNN-TL_OASIS-2_, and CNN-TL_OASIS-3_, respectively.

### 3.2. Ablation Study of TL

**Figure 6:**
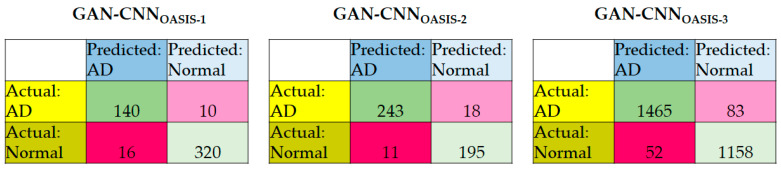
Confusion matrices of the GAN-CNN_OASIS-1_, GAN-CNN_OASIS-2_, and GAN-CNN_OASIS-3_.

Table 4 summarizes the performance evaluation of GAN-CNN for OASIS-1, OASIS-2, and OASIS-3. It is noted the with GAN, 5-fold cross-validation can be resumed. Figure 6 summarizes the confusion matrices of the models using three datasets. The key observations are summarized as follows.

- The ranking, in descending order, of the sensitivity of the three models is GAN-CNN_OASIS-3_, GAN-CNN_OASIS-2_, GAN-CNN_OASIS-1_; for the specificity and accuracy, the ranking is GAN-CNN_OASIS-3_, GAN-CNN_OASIS-1_, GAN-CNN_OASIS-2_;
- There are deviations between the accuracies of a single class because of the number of available ground truth data in each class and the characteristics (and thus the difficulty in AD detection) of the class. The average deviations are 3.07%, 1.65%, and 1.13% for GAN-CNN_OASIS-1_, GAN-CNN_OASIS-2_, and GAN-CNN_OASIS-3_, respectively;
- The deviations between the sensitivity and specificity are 2.15%, 1.65%, and 1.16% for GAN-CNN_OASIS-1_, GAN-CNN_OASIS-2_, and GAN-CNN_OASIS-3_, respectively.

## 4. Results Analysis and Comparison

### 4.1. Performance Evaluation of the Proposed Method

**Figure 7:**
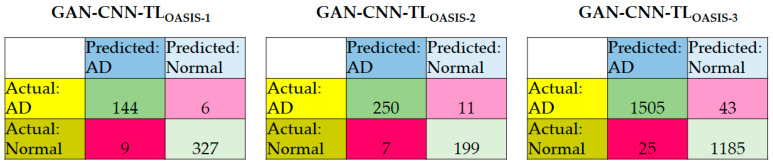
Confusion matrices of the GAN-CNN-TL_OASIS-1_, GAN-CNN-TL_OASIS-2_, and GAN-CNN-TL_OASIS-3_.

The accuracy of a single class, sensitivity, specificity, and accuracy of the average of the results from fivefold cross-validation are recorded in the performance evaluation of the GAN-CNN-TL for OASIS-1, OASIS-2, and OASIS-3 models, as shown in Table 5. Figure 7 summarizes the confusion matrices of the models using three datasets. It is noted that these models have been designed for validation in OASIS-1, OASIS-2, and OASIS-3, respectively. Given that there is one class related to normal participants, the specificity is equal to the accuracy of Class 0. In regard to the sensitivity, it is the weighted average of the accuracies of Class 1, Class 2, and Class 3.

Here are the key observations of the results:

- In all models, the specificity is higher than the sensitivity due to the main reason of majority class (Class 0). The accuracy is in between the sensitivity and specificity as the weighted accuracy;
- The ranking, in descending order, of the sensitivity of the three models is GAN-CNN-TL_OASIS-3_, GAN-CNN-TL_OASIS-2_, GAN-CNN-TL_OASIS-1_; for the specificity and accuracy, the ranking is GAN-CNN-TL_OASIS-3_, GAN-CNN-TL_OASIS-1_, GAN-CNN-TL_OASIS-2_;
- There are deviations between the accuracies of a single class because of the number of available ground truth data in each class and the characteristics (and thus the difficulty in AD detection) of the class. The average deviations are 3.29%, 1.67%, and 0.65% for GAN-CNN-TL_OASIS-1_, GAN-CNN-TL_OASIS-2_, and GAN-CNN-TL_OASIS-3_, respectively;
- The deviations between the sensitivity and specificity are 1.35%, 0.83%, and 0.62% for GAN-CNN-TL_OASIS-1_, GAN-CNN-TL_OASIS-2_, and GAN-CNN-TL_OASIS-3_, respectively.

### 4.2. Results Comparison between Our Work and Existing Works

In regard to the performance comparison between our work and existing works [11,12,13,14,15,16,17,18,19,20,21,22], the dataset, class and sample size, features, algorithm, type of cross-validation, sensitivity, specificity, and accuracy are summarized in Table 6. The discussion is presented based on each dataset and each perspective.

#### 4.2.1. OASIS-1

- Class and sample size: Our work and [12] utilized the full set of OASIS-1 for four-class AD detection. Work [11] merged Class 2 and Class 3 as one class, i.e., mild/moderate AD. The remaining two works [13,14] considered a binary AD detection. Four-class AD detection is desired to better reflect the nature of the categorization of different types of AD;
- Features and algorithms: Work [11] separated the feature extraction and AD detection into two parts using two algorithms. Our work and [12,13,14] formulated the feature extraction and AD detection with one algorithm, which is known as automatic feature extraction;
- Type of cross-validation: Work [13] did not employed cross-validation that may result in insufficiency in hyperparameter tuning and evaluation of the model overfitting. Twofold cross-validation was adopted in our work and Fivefold cross-validation was used in [12,14]. Tenfold cross-validation was used in [11], nevertheless, it was inappropriately formulated using a 80:20 ratio between the training and testing datasets;
- Sensitivity: The sensitivity was reported only in our work and [13]. Our work improved the sensitivity by 43.3% even when a four-class AD detection was formulated;
- Specificity: The specificity was reported only in our work and [13]. Our work improved the sensitivity by 24.6%, even when a four-class AD detection was formulated;
- Accuracy: Our work improved the accuracy by 21–33.0% compared with [12,13,14]. Comparing the result with [11], our work was 2.21% less accurate, however, we have formulated the AD detection model as four-class, in contrast to the three-class model in [11]. The result in [11] may not be fully reflective of reality as the 10-fold cross-validation was inappropriately defined in the ratio between training and testing datasets.

#### 4.2.2. OASIS-2

- Class and sample size: Only our work utilized the full set of OASIS-2 for four-class AD detection. The remaining works [15,16,17,18] formulated the problem as binary AD detection, where the detector only determines if the participant suffers from AD (without the information of the severity);
- Features and algorithms: Works [15,17] separated the feature extraction and AD detection into two parts using two algorithms. Our work and [16,18] formulated the feature extraction and AD detection with one algorithm;
- Type of cross-validation: Work [13] did not employ cross-validation, which may result in insufficiency in hyperparameter tuning and evaluation of the model overfitting. Threefold cross-validation was adopted in our work, whereas fivefold cross-validation was used in [12,14]. Tenfold cross-validation was used in [11], nevertheless, it was inappropriately formulated with a 80:20 ratio between training and testing dataset;
- Sensitivity: The sensitivity was reported only in our work and [15]. Our work improved the sensitivity by 8.96%, even when a four-class AD detection is formulated;
- Specificity: The specificity was reported only in our work and [15]. Our work decreased the specificity by 3.2% using a 4-class AD detection. Taking sensitivity into consideration, a biased detection model was built in [15];
- Accuracy: Our work improved the accuracy by 1.80–40.1% compared with [15,17,18]. Comparing the result with [16], our work was 0.622% less accurate, however, we have formulated the AD detection model as four-class, in contrast to the two-class model and smaller size of the dataset in [16].

#### 4.2.3. OASIS-3

- Class and sample size: Our work and [20,21] utilized the full set of OASIS-3 for four-class AD detection. Work [19] formulated a binary AD detection model, whereas work [20] designed a one-class AD detection model;
- Features and algorithms: One work [21] separated the feature extraction and AD detection into two parts using two algorithms. Our work and [19,20,22] formulated the feature extraction and AD detection with one algorithm;
- Type of cross-validation: The existing works [19,20,21,22] did not employ cross-validation. Fivefold cross-validation was adopted in our work;
- Sensitivity: The sensitivity was reported only in our work and [20]. Our work improved the sensitivity by 16.5%;
- Specificity: The specificity was reported only in our work and [20]. Our work improved the specificity by 7.11%;
- Accuracy: our work improved the accuracy by 7.97–31.0% compared with [19,20,21,22].

## 5. Conclusions

Smart health, as one of the essential areas in smart city visions, requires advanced technology to improve the existing healthcare systems. In this paper, automatic diagnosis of AD using a machine learning model is believed to relieve the workload of medical staff and increase the accuracy of medical diagnoses. This paper proposes a GAN-CNN-TL algorithm that provides the advantages of additional data generation, reduction of biased detection model, automatic feature extraction, and enhancement in hyperparameter tuning. Performance evaluation and analysis using three benchmark (OASIS-series) datasets revealed the effectiveness of the proposed method that increases the accuracy of the detection model by 2.85–3.88%, 2.43–2.66%, and 1.8–40.1% in the ablation study of GAN and TL, as well as the comparison with existing works, respectively. The analysis also revealed that the proposed algorithm resolves the limitations of the existing works.

## Figures and Tables

**Table 1:** Constituents of OASIS-1, OASIS-2, and OASIS-3.

		OASIS-1	OASIS-2	OASIS-3
Number of participants		416	150	1098
Sample size of class label	Class 0: Normal	336	206	1210
	Class 1: very mild AD	70	123	516
	Class 2: Mild AD	28	41	262
	Class 3: Moderate AD	2	3	180

**Table 2:** Imbalanced ratios across different classes of OASIS-1, OASIS-2, and OASIS-3.

	Imbalanced Ratio		
Class Label	OASIS-1	OASIS-2	OASIS-3
Class 0: Normal	N/A	N/A	N/A
Class 1: very mild AD	4.8:1	1.67:1	2.34:1
Class 2: Mild AD	12:1	5.02:1	4.62:1
Class 3: Moderate AD	168:1	68.7:1	6.72:1

**Table 3:** Performance evaluation of the CNN-TL for OASIS-1, OASIS-2, and OASIS-3.

Model	Accuracy of a Single Class (%)	Sensitivity (%)	Specificity (%)	Accuracy (%)
CNN-TL_OASIS-1_	Class 0: 94.6	91.0	94.6	93.8
	Class 1: 92.9			
	Class 2: 89.3			
	Class 3: 50			
CNN-TL_OASIS-2_	Class 0: 93.7	91.6	93.7	92.8
	Class 1: 93.5			
	Class 2: 87.8			
	Class 3: 66.7			
CNN-TL_OASIS-3_	Class 0: 95.3	94.1	95.3	94.8
	Class 1: 94.6			
	Class 2: 93.5			
	Class 3: 92.8			

**Table 4:** Performance evaluation of the GAN-CNN for OASIS-1, OASIS-2, and OASIS-3.

Model	Accuracy of a Single Class (%)	Sensitivity (%)	Specificity (%)	Accuracy (%)
GAN-CNN_OASIS-1_	Class 0: 95.2	93.4	95.2	94.6
	Class 1: 94.3			
	Class 2: 92.9			
	Class 3: 90			
GAN-CNN_OASIS-2_	Class 0: 94.7	93.1	94.7	93.8
	Class 1: 93.5			
	Class 2: 92.7			
	Class 3: 93.3			
GAN-CNN_OASIS-3_	Class 0: 95.7	94.6	95.7	95.1
	Class 1: 95.1			
	Class 2: 94.6			
	Class 3: 94.2			

**Table 5:** Performance evaluation of the GAN-CNN-TL for OASIS-1, OASIS-2, and OASIS-3.

Model	Accuracy of a Single Class (%)	Sensitivity (%)	Specificity (%)	Accuracy (%)
GAN-CNN-TL_OASIS-1_	Class 0: 97.3	96.0	97.3	96.9
	Class 1: 97.1			
	Class 2: 95.8			
	Class 3: 90			
GAN-CNN-TL_OASIS-2_	Class 0: 96.6	95.8	96.6	96.1
	Class 1: 95.9			
	Class 2: 95.9			
	Class 3: 93.3			
GAN-CNN-TL_OASIS-3_	Class 0: 97.9	97.3	97.9	97.5
	Class 1: 97.5			
	Class 2: 97.3			
	Class 3: 97.0			

**Table 6:** Performance comparison between our work and existing works.

Work	Dataset	Class and Sample Size	Features	Algorithms	Type of Cross-Validation	Sensitivity (%)	Specificity (%)	Accuracy (%)
[11]	OASIS-1	Healthy: 316 Very mild AD: 70 Mild/moderate AD: 30	Gradient boosted random forest	ResNet-50	10-fold (with an inappropriate 80:20 ratio)	N/A	N/A	98.99
[12]	OASIS-1	Healthy: 336 Very mild AD: 70 Mild AD: 28 Moderate AD: 2	BrainNet3D		5-fold	N/A	N/A	80
[13]	OASIS-1	Healthy: 41 AD: 37	Adversarial autoencoder		No	67	78	72.8
[14]	OASIS-1	Healthy: 100 AD: 100	M-Net-axial_32		5-fold	N/A	N/A	74.9
**Our Work**	OASIS-1	Healthy: 336 Very mild AD: 70 Mild AD: 28 Moderate AD: 2	GAN-CNN-TL		2-fold	96	97.2	96.8
[15]	OASIS-2	Healthy: 206 AD: 167	Boruta	Deep neural network	No	88.2	100	94.7
[16]	OASIS-2	Healthy: 72 AD: 64	CNN		No	N/A	N/A	97
[17]	OASIS-2	Healthy: 206 AD: 167	Subject ID, clinical dementia ratio, mini-mental state examination, age, magnetic resonance delay, and normalized whole brain volume	SVM	No	N/A	N/A	68.8
[18]	OASIS-2	Healthy: 41 AD: 41	Voxel-size independent neural network		No	N/A	N/A	88.2
**Our Work**	OASIS-2	Healthy: 206 Very mild AD: 123 Mild AD: 41 Moderate AD: 3	GAN-CNN-TL		3-fold	96.1	96.8	96.4
[19]	OASIS-3	Healthy: 100 AD: 100	Gray level co-occurrence matrix and CNN		No	N/A	N/A	90.3
[20]	OASIS-3	Healthy: 1210 Very mild AD: 516 Mild AD: 262 Moderate AD: 180	Ensemble learning of Inception-v3, DenseNet121, ResNet50, and ResNet18		No	83.5	91.4	87.9
[21]	OASIS-3	Healthy: 1210 Very mild AD: 516 Mild AD: 262 Moderate AD: 180	vertex-based graph-CNN	RNN	No	N/A	N/A	82.6
[22]	OASIS-3	Healthy: 1210	Deep convolutional generative adversarial network		No	N/A	N/A	74.4
**Our Work**	OASIS-3	Healthy: 1210 Very mild AD: 516 Mild AD: 262 Moderate AD: 180	GAN-CNN-TL		5-fold	97.3	97.9	97.5

## Data Availability

Not applicable.
